# The Regulatory Role of Long Non-Coding RNAs in the Development and Progression of Osteoporosis

**DOI:** 10.3390/ijms26094273

**Published:** 2025-04-30

**Authors:** Rogelio F. Jiménez-Ortega, Diana I. Aparicio-Bautista, Adriana Becerra-Cervera, Alejandra I. Ortega-Meléndez, Nelly Patiño, Berenice Rivera-Paredez, Alberto Hidalgo-Bravo, Rafael Velázquez-Cruz

**Affiliations:** 1 Clínica Integral Universitaria (CIU), Universidad Estatal del Valle de Ecatepec (UNEVE), Ecatepec de Morelos 55210, Mexico; rogeliofrank.jimenez@uneve.edu.mx; 2 Programa Investigadoras e Investigadores, Consejo Mexiquense de Ciencia y Tecnología (COMECYT), Toluca 50120, Mexico; 3 Laboratorio de Genómica del Metabolismo Óseo, Instituto Nacional de Medicina Genómica (INMEGEN), Mexico City 14610, Mexico; daparicio@inmegen.gob.mx (D.I.A.-B.); abecerra@inmegen.edu.mx (A.B.-C.); 4 Secretaría de Ciencias, Humanidades, Tecnología e Innovación (SECIHTI), Ciudad de México 03940, Mexico; 5 Unidad Académica de Ciencias de la Salud, Universidad ETAC Campus Coacalco, Coacalco de Berriozábal 55700, Mexico; alejandra.ortega@universidadetac.edu.mx; 6 Unidad de Citometría de Flujo (UCiF), Instituto Nacional de Medicina Genómica (INMEGEN), Mexico City 14610, Mexico; lnpatino@inmegen.gob.mx; 7 Centro de Investigación en Políticas, Población y Salud, Facultad de Medicina, Universidad Nacional Autónoma de México, Mexico City 04510, Mexico; bereriveraparedez7@gmail.com; 8 Departamento de Medicina Genómica, Instituto Nacional de Rehabilitación (INR), Mexico City 14389, Mexico; dr_genetica@yahoo.com

**Keywords:** osteoporosis, lncRNA, osteoclasts, osteoblasts, osteocytes, bone mineral density

## Abstract

Osteoporosis (OP) is a disease affecting bone metabolism, characterized by low bone mineral density and the deterioration of the bone microarchitecture, leading to increased bone fragility and risk of fracture. OP mainly results from alterations in the balance between osteoclast-mediated bone resorption and osteoblast-mediated bone formation. Currently, there are several molecular mechanisms underlying the development of OP that are not entirely clear. One such mechanism is the role of long non-coding RNAs, which are key regulators of gene expression through various mechanisms. In the last decade, it has been shown that these molecules participate in multiple biological processes and play essential roles in the pathogenesis of different diseases. In this review, we address recent advances on the relationship of long non-coding RNAs with OP, mainly over their regulatory functions during osteoclastogenesis and osteogenesis. Furthermore, we analyze their potential application as clinical or therapeutic resources focused on OP.

## 1. Introduction

Osteoporosis (OP) is a systemic and progressive skeletal disorder affecting approximately 200 million people worldwide every year [1]. OP is a major cause of morbidity and mortality and has a high impact on healthcare systems. The medical, economic, and social burden of fragility fractures remains high in most developed countries and is increasing substantially in other countries [2]. For the current year (2025), fracture costs are estimated reach USD 25.0 billion in the United States and USD 37.0 billion in the European Union. Meanwhile, in Latin American countries, such as Mexico and Argentina, costs could reach up to USD 442.0 million and USD 298.0 million, respectively [3,4].

OP occurs due to an imbalance in the bone remodeling process. This imbalance is characterized by the increased activity of osteoclasts (bone-tissue-resorptive cells) and low activity of osteoblasts (bone-tissue-forming cells), leading to bone fragility and susceptibility to fractures [5]. Osteoclasts originate from hematopoietic stem cells, giving rise to mononuclear monocyte/macrophage lineage precursors. Afterward, these cells fuse and form multinucleated giant cells, a process called osteoclastogenesis.

On the other hand, osteoblasts originate from mesenchymal stem cells located at the bone marrow stroma, and they are responsible for the synthesis of bone matrix and its subsequent mineralization [6]. OP occurs predominantly in postmenopausal women, and although there are different diagnostic tools aimed for evaluating the risk of fracture, these have limitations, restricting their use in clinical practice. Therefore, there is a need to find new methods for early diagnosis and new OP treatment guidelines [7].

Recent studies have reported that osteoclastogenesis and osteoblastogenesis are regulated by genetic and epigenetic factors, including non-coding RNAs (ncRNAs) [8]. ncRNA are molecules that can be classified according to their length, as short ncRNAs, less-than-200-nucleotides-in-length piwi-interacting RNAs (piRNAs), interfering RNAs (iRNAs), small nuclear RNAs (snRNAs), and microRNAs (miRNAs), among others. On the other hand, non-coding transcripts larger than 200 nucleotides are called long non-coding RNAs (lncRNAs) [9]. LncRNAs have diverse regulatory functions, including the regulation of transcription through protein–RNA interaction, chromatin remodeling, translation regulation, protein modification, and miRNA sponges. Alterations in the expression profiles of lncRNAs have been associated with adverse consequences on cell function and ultimately human health. LncRNA have been proposed as potential biomarkers of multiple metabolic diseases, including those affecting bone metabolism [10]. This review describes the biogenesis, regulation, and function of lncRNAs in bone metabolism and how expression changes can lead to developing bone diseases such as OP.

## 2. Long Non-Coding RNAs: Their Role in Bone Metabolism Diseases

LncRNAs are molecules recognized as an emerging class of transcripts encoded in over 80% of the human genome. However, despite their abundance, their biological functions are not elucidated yet [11]. Initially, lncRNAs were considered transcriptional “junk” or “noise”; however, in recent years, several studies have revealed that they have crucial roles in various physiological and pathological processes; they are key gene expression regulators controlling virtually all cellular processes [12]. The dysregulation of lncRNAs has been associated with OP development and progression by regulating the expression of genes involved in the cell cycle, proliferation, apoptosis, and cell invasion [1,13].

### 2.1. Classification of LncRNAs

LncRNAs are classified based on their genomic locations and their orientations, and five categories are recognized: sense, antisense, bidirectional, intronic, and intergenic [14]. LncRNA sequences are poorly conserved between species; on the contrary, their promoter sequences remain highly conserved [15,16,17,18]. Among their functions, lncRNAs can bind to specific protein motifs to regulate their activity and cellular localization, as well as form protein substructures or complexes and influence specific post-transcriptional processes [19]. The origin of lncRNAs is still not fully understood. However, several studies suggest that more than two-thirds of lncRNA transcripts may contain transposable elements (TEs) of the genomes [20,21]. The transcription of lncRNAs is initiated from divergent promoters depending on the direction of the mRNA; some lncRNAs are transcribed in antisense from the promoters of coding genes. Transcription in the divergent direction is enhanced by proteins of the SWitch/sucrose non-fermentable (SWI/SNF) complex and repressed by chromatin assembly factor (CAF)-1. The appearance of U1 and polyadenylation signals differ on both sides of bidirectional promoters, promoting mRNA splicing in the sense direction and their cleavage and polyadenylation in the divergent antisense direction. LncRNAs are variably located in different chromosome regions, and we can find them interacting with chromatin, the nucleoplasm, and the cytoplasm [22].

### 2.2. Mechanisms of Action of LncRNAs

In general, the functions of lncRNAs depend on their subcellular localizations. Five archetypal molecular functions have been characterized: (1) lncRNAs as signals, are expressed at specific sites within cells in response to different stimuli that participate in transcriptional regulation and chromatin remodeling [23,24,25]; (2) lncRNA as a decoy, which sequesters regulatory factors such as miRNAs and transcription factors, repressing gene transcription [24,26]; (3) lncRNAs as guides, they function as direct modifiers of chromatin complexes and proteins such as ribonucleoproteins (RNPs), which are stimulated by RNA–RNA, RNA–protein, and RNA–DNA interactions [27]); (4) lncRNAs as scaffolds, which play an essential structural role in the assembly of multiprotein complexes, including those composed of short-lived RNPs once fully assembled, RNP complexes can either suppress or activate transcription [28]; and (5) lncRNAs as enhancers, which send molecular signals to initiate transcriptional regulation in response to different stimuli. Finally, lncRNAs may have additional regulatory functions, such as protein trafficking and signaling [29].

### 2.3. The Role of LncRNAs in Bone Resorption

Osteoclasts play an essential role in bone metabolism, and their overactivation is associated with the development of postmenopausal OP. Several lncRNA are involved in osteoclast differentiation [30]; some of them have been identified as negative regulators of this process. For example, the abundances of lncRNA GAS5 and miR-21 were found to be inversely correlated in plasma derived from patients with OP. In osteoclasts, the overexpression of GAS5 lead to the suppression of miR-21. In addition, the overexpression of GAS5 promotes osteoclast apoptosis whereas the overexpression of miR-21 suppresses it. These findings suggest a regulating network involving GAS5 and miR-21 with a regulatory effect on osteoclastogenesis [31]. Similarly, TUG overexpression suppressed osteoclastogenesis induced by RANKL and M-CSF by potentiating the degradation of V-maf musculoaponeurotic fibrosarcoma oncogene homolog B (*MafB*), considered an anti-osteoclastogenic factor, therefore increasing bone resorption [32,33]. In line, lncRNA DANCR reduced osteoclastogenesis and compressive-force-induced root resorption through the expression of Jagged1 in periodontal ligament cells whereas lncRNA Bmncr decreased the progression of osteoporosis by inhibiting RANKL-induced osteoclastic differentiation. While other lncRNAs function as positive regulators of bone resorption, for example, the overexpression of lncRNA cancer susceptibility 11 (CASC11) led to upregulated TNF-α in osteoclasts, potentially exacerbating bone loss in postmenopausal OP in a study [32].

In the same way, Receptor Tyrosine Kinase 1 like orphan receptor (ROR1) regulates the Hippo-YAP pathway, promoting osteoclast differentiation and bone metastasis through the recruitment of the LLGL2-MAYA-NSUN6 protein–RNA complex [34]. LncRNA_MIRG induces osteoclastogenesis and bone resorption in osteoporosis via the downregulation of miR-1897 [35]. In BMSCs, ENSG00000257764.2-miR-106 a-5p-TIMP2 may play a central role in osteoclastic differentiation [36].

The evidence suggests that lncRNAs may act as positive or negative regulators of osteoclast formation, differentiation, and apoptosis, making them potential therapeutic targets for inhibiting excessive bone resorption. As more mechanisms of action of lncRNAs are discovered, targeted therapies explicitly regulating osteoclast activity may be developed, which could transform the treatment of bone disorders such as OP. Therefore, more research is required to fully understand their role in human models and establish the feasibility of these approaches in clinical practice. Table 1 summarizes the studies analyzing the role of lncRNAs in osteoclastogenesis in cell lines and animal models [37,38,39,40,41,42,43,44,45,46,47,48,49,50].

### 2.4. The Role of LncRNAs in Bone Formation

The bone formation process requires the precise coordination of osteoblast differentiation, matrix mineralization, and bone remodeling. In recent years, the influence of lncRNAs has extended to a wide range of cellular processes, including cell cycle regulation, cell proliferation, metastasis, immunobiological responses, and cell differentiation [51]. A study by Jiang et al. (2019) [52] reported a significant increase in the expression levels of lncRNA DANCR in the serum of patients with fractures. Meanwhile, in vitro experiments in cell lines demonstrated that the intervention of DANCR with siRNA promotes the proliferation and differentiation of the osteoblast cell line MC3T3-E1. In addition, they showed that DANCR could promote apoptosis and proliferation through the Wnt/β-catenin signaling pathway activated in osteoblasts. Therefore, DANCR inhibition may encourage osteoblast proliferation and differentiation, making it a potential therapeutic target for treating fragility fractures. Recently, Wang et al. (2020) [53] reported the interaction between DANCR/miR-320a/*CTNNB1* in bone marrow mesenchymal stem cells (BMSCs) from postmenopausal women, where the aberrant expression of *CTNNB1* (β-catenin) is associated with alterations in Wnt/β-catenin signaling. In this study using bioinformatics tools, the authors reported that both miR-320a and DANCR present binding sites and act directly on *CTNNB1*, inhibiting osteogenic differentiation. Through in vitro and in vivo methods, the authors demonstrated that the expression levels of DANCR and miR-320a were notably higher in cells from OP patients than in healthy controls.

In contrast, the expression of *CTNNB1* remained downregulated. On the other hand, during the osteogenic differentiation process, the expression levels of DANCR and miR-320a were attenuated in BMSCs while *CTNNB1* mRNA and protein levels increased significantly. Another study by Wei et al. (2017) [54] analyzed the relationship between lncRNA HOTAIR and miR-17-5p in osteogenic differentiation and proliferation in the non-traumatic osteonecrosis of the femoral head (ONFH). Elevated HOTAIR expression and low miR-17-5p levels were observed in BMP-2-induced mesenchymal stem cells (MSCs) from patients with non-traumatic ONFH and osteoarthritis (OA). Additionally, the downregulation of HOTAIR induced by si-HOTAIR resulted in the increased expression of miR-17-5p and decreased expression of SMAD Family Member 7 (*SMAD7*), a target gene of this miRNA. Osteogenic differentiation markers such as *RUNX2*, *COL1A1*, and *ALP* were also increased by si-HOTAIR. However, this effect was counteracted by the miR-17-5p inhibitor or by *SMAD7* overexpression, suggesting that HOTAIR could play a key role in regulating osteogenic differentiation and proliferation by modulating miR-17-5p and *SMAD7* in non-traumatic ONFH.

On the other hand, Li et al. (2019) [55] observed a significant increase in the expression of the lncRNA_MEG3 in the sera of patients with fragility fractures. They found that downregulating MEG3 using siRNAs promoted the proliferation and differentiation of osteoblasts, which was mediated by the Wnt/β-catenin signaling pathway. These results highlight MEG3 as a potential therapeutic target to accelerate fracture recovery.

A study conducted by Che et al. (2020) [56] evaluated the expression levels and interactions between lncRNA_HLA complex group 18 (HCG18) and miR-30a-5p in bone marrow mesenchymal stem cells (BMSCs) derived from mouse models and OP patients. The results showed an overexpression of HCG18 while the expression of miR-30a-5p was decreased in BMSCs-derived OP patients, indicating a negative correlation. A contrary effect was observed during the differentiation of BMSCs to osteoblasts; the expression of *HCG18* was downregulated and the expression of miR-30a-5p was significantly upregulated. The overexpression of HCG18 was able to reverse the osteogenesis-induced upregulation of miR-30a-5p expression while the suppression of HCG18 further promoted miR-30a-5p expression. *NOTCH1* was a target gene of miR-30a-5p, and the upregulation of *NOTCH1* reversed the inhibitory effect of miR-30a-5p on the osteogenic differentiation of BMSCs. These findings suggest that HCG18 inhibited the OP-induced osteogenic differentiation of BMSCs through the miR-30a-5p/*NOTCH1* axis, positioning HCG18 as a key regulator of bone formation.

Finally, Han et al. (2022) [57] analyzed the role of lncRNA small nucleolar RNA host gene 5 (*SNHG5*), Yin yang 1 (*YY1*) gene, miR-212-3p, and growth differentiation factor 5 (*GDF5*) in the osteogenic differentiation of human BMSCs, in vitro and in vivo. The authors observed that *SNHG5* expression was upregulated during BMSC osteogenesis and its suppression inhibited osteogenic differentiation while its overexpression promoted it. Furthermore, the transcription factor YY1 directly bound to the promoter region of *SNHG5* and regulated its expression to promote osteogenesis. Dual-luciferase reporter assays confirmed that *SNHG5* acts as a sponge for miR-212-3p, which targets *GDF5*, activating the Smad1/5/8 phosphorylation. These results demonstrate that miR-212-3p inhibited osteogenesis whereas *GDF5* promotes it. This study suggests that the *YY1*/*SNHG5*/miR-212-3p/*GDF5*/*Smad* signaling pathway is involved in osteogenic differentiation and is a potential target for treating bone loss.

The information revealed that lncRNAs directly influence the regulation of osteoblast differentiation by intervening in various signaling pathways, such as those of Wnt/β-catenin and miR-17-5p/*SMAD7*. This demonstrates that lncRNAs are crucial players in the control of bone health. Several studies suggest that the modulation of lncRNAs could be a therapeutic strategy to enhance osteogenic differentiation, accelerate fracture recovery, or even prevent bone loss. Table 2 describes the studies analyzing the role of lncRNA implicated in osteoblastogenesis in humans, cell lines, and animal models [58,59,60,61,62,63,64,65,66,67,68,69,70,71,72,73].

### 2.5. The Role of LncRNA in Osteocytes

Although osteoblasts and osteoclasts are key players in bone biology, the osteocytes are the primary cells that constitute 90–95% of bone tissue. However, their involvement in bone homeostasis is often underestimated, and their regulatory mechanisms remain unclear. Currently, there have been few reports about the involvement of lncRNAs in osteocytes. A study by Fu et al. (2019) [74] identified lncRNA_HOXA transcript antisense RNA, myeloid-specific 1 (*HOTAIRM1*), as a critical regulator of osteogenesis in human MSCs. *HOTAIRM1* significantly inhibited calcium deposition and alkaline phosphatase activity in MSCs and positively modulated JNK and c-Jun activity, which are key modulators of the osteogenic differentiation of MSCs to osteocytes.

**Figure 1:**
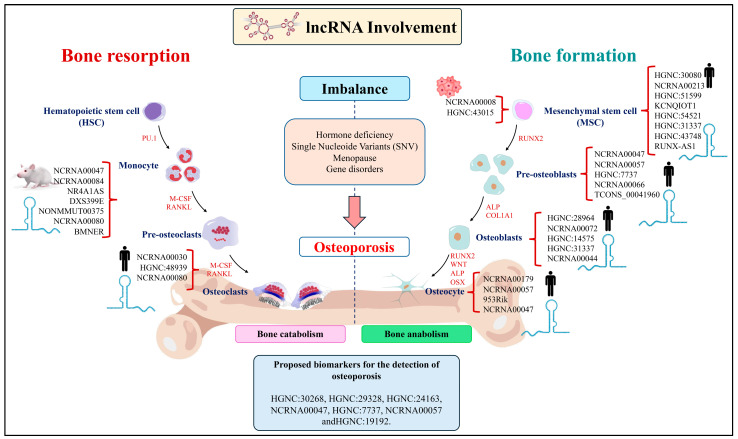
The lncRNAs that act as inhibitors or promoters of the bone resorption and formation process are shown. The lncRNAs are key transcriptional and translational regulators that act as modulators of processes such as chromatin remodeling, ligands of activators/repressors of genetic promoters, transcriptional modulators of other regulatory RNAs, and endogenous RNAs competitively.

Another study by Yu et al. (2021) [75] analyzed the mechanism of the lncRNA small nucleolar RNA host gene 1 (*SNHG1*) in bone differentiation and angiogenesis in OP development in mouse serum and femoral tissue samples. An increase in *SNHG1* expression promoted a decrease in miR-181c-5p expression, activating Wnt3a/β-catenin signaling by upregulating secreted frizzled-related protein 1 (SRFP1), a negative regulator of osteoblast and osteocyte survival in humans. Additionally, *SNHG1* inhibition promoted the osteogenic differentiation of BMSCs by increasing miR-181c-5p expression while *SNHG1* overexpression promoted osteoclastic differentiation and inhibited angiogenesis. This indicates that *SNGH1* enhances the expression of *SFRP1* by absorbing miR-181c-5p and regulates bone remodeling and angiogenesis. Notably, *SFRP1* is an antagonist of the Wnt/β-catenin signaling pathway and can inhibit the downstream transduction of Wnt. The Wnt/β-catenin pathway promotes bone mineralization by stimulating osteoblast proliferation, differentiation, and survival, and it also inhibits osteoclast differentiation and osteocyte activity. Therefore, the authors suggest that *SNHG1* may contribute to the progress of OP by suppressing osteogenesis and thus could be considered as a potential therapeutic target for the treatment of OP. On the other hand, Arai et al. (2023) reported that lncRNA953Rik is involved in osteogenic differentiation through the inhibition of Osterix, resulting in the suppression of osteoblast-to-osteocyte differentiation in a mouse model [76]. Given that osteocytes play a significant role in maintaining bone integrity, further research is necessary to understand the role of lncRNA during bone metabolism and its regulatory mechanisms. Some of the lncRNAs function as inhibitors or promoters of bone tissue resorption and formation (Figure 1).

## 3. LncRNAs Involved in the Development of Osteoporosis

**Figure 2:**
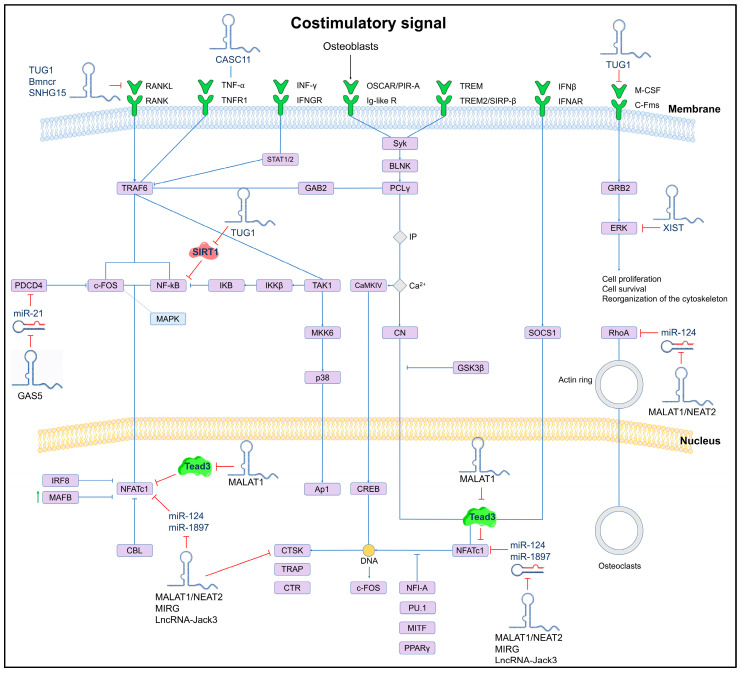
Schematic of the osteoclastogenesis signaling pathway and its lncRNA-induced regulation. The regulatory effect of lncRNAs on miRNAs and their downstream target genes is shown. LncRNAs can directly (solid lines) or indirectly (dashed lines) inhibit key genes involved in osteoclast differentiation.

Currently, few lncRNAs have been associated with OP, and one of the most reported is MALAT1, which is abundantly expressed in normal tissues. Targeted inactivation and gene rescue experiments have identified that MALAT1 plays a key role in suppressing lung metastasis in breast cancer. In mouse and human models, the downregulation of MALAT1 promotes osteoclastogenesis through its binding to the protein Tead3, a member of the Tead family specific to macrophages and osteoclasts, which binds to the master transcription factor Nfatc1, a key regulator of osteoclastogenesis, resulting in the inhibition of Nfatc1-mediated gene transcription and osteoclast differentiation. It is worth mentioning that, according to the single-cell transcriptome analysis of bone samples, the reduced expression of MALAT1 in osteoclast precursors and osteoclasts is associated with the development of OP and metastatic lesions, so this lncRNA is considered a protective molecule against OP and bone metastasis [77]. The mechanism of action of MALAT1 and other lncRNAs involved in osteoclastogenesis is shown in Figure 2.

**Figure 3:**
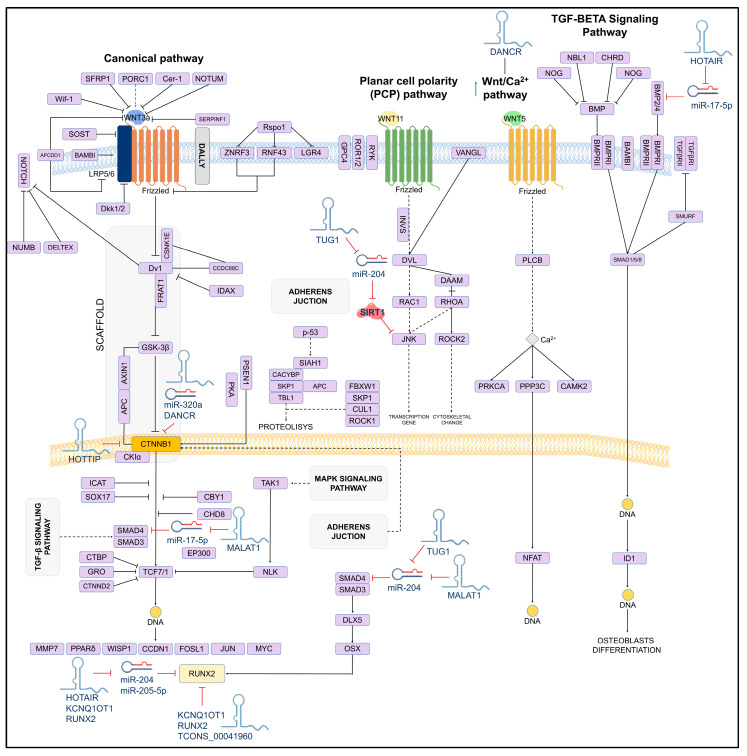
Schematic of the osteoblastogenesis signaling pathway and its lncRNA-induced regulation. The regulatory role of lncRNAs on miRNAs and target genes is shown. LncRNAs can directly (solid lines) or indirectly (dashed lines) inhibit key genes for osteoblast differentiation.

On the other hand, studies have reported that in human mesenchymal stem cells, the lncRNA_TUG1 acts as a positive regulator of osteoblastogenesis by downregulating miR-204, which in turn inhibits *SIRT1*, which is a crucial mediator of osteoblast function. *SIRT1* interacts with proteins such as NF-kB, a promoter of osteoclastogenesis. A similar mechanism has been observed with *JNK1*, a critical factor for osteoblasts differentiation, which is also regulated by miR-204 [78]. The mechanism of action of TUG1 and other lncRNAs involved in osteoblastogenesis is shown in Figure 3.

## 4. Perspectives of Clinical Applications of LncRNAs

As mentioned throughout this review, lncRNAs play a key role in essential processes related to the development of bone-related diseases, including OP. Some lncRNA have been suggested as biomarkers with potential diagnostic, prognostic, and therapeutic targets. For example, lncRNA-RAB37, lncRNA-ZNF529, and lncRNA-BEGAIN were differentially expressed in peripheral blood mononuclear cells (PBMCs) in normal postmenopausal women and those with OP. The results of the bioinformatics analysis indicated a close association with postmenopausal OP. The area under the ROC curve (AUC) analysis showed a high degree of sensitivity and specificity (AUC > 96%), suggesting that these lncRNAs could be potential biomarkers for diagnosing postmenopausal OP [69]. Recently, lncRNA-MALAT1 has been shown to promote osteogenesis under different health conditions and may play a key role in postmenopausal OP. In a study, the expression levels of lncRNA-MALAT1 were evaluated in the plasma of postmenopausal OP patients and found to be significantly lower than in healthy subjects. Plasma expression levels of lncRNA-MALAT1 were positively associated with total hip, femoral neck, and lumbar spine BMD in postmenopausal women with OP. An AUC analysis was used to evaluate the potential diagnostic value of lncRNA-MALAT1 with respect to Genant’s semi-quantitative (GSQ) criteria used to assess vertebral deformity and fracture. These data suggest that lncRNA-MALAT1 is a potential biomarker targeting the timely diagnosis of postmenopausal OP [79]. LncRNA-NEF, an oncogene involved in cancer biology and postmenopausal OP, was found to be downregulated in the plasma samples of OP patients while IL-6 was upregulated compared to healthy controls. The diagnostic significance of plasma concentrations of lncRNA-NEF and IL-6 for postmenopausal OP was evaluated, with AUC values of 89.19% and 77.43%, respectively. The authors concluded that the expression of lncRNA-NEF decreases postmenopausal OP and correlates with the treatment duration and recurrence rate. Therefore, this lncRNA could be a potential target for treating postmenopausal OP [80].

The lncRNA-SNHG1 found in the plasma of pre- and postmenopausal women with OP showed significantly lower expression levels than normal controls. A 6-year follow-up in postmenopausal women revealed that plasma lncRNA-SNHG1 levels were decreased in women with postmenopausal OP but not in healthy postmenopausal women. Plasma lncRNA-SNHG1 levels measured in plasma 12 months before diagnosis could effectively differentiate patients with postmenopausal OP from healthy controls. It was observed that plasma lncRNA-SNHG1 was significantly upregulated after OP-targeted treatment. Therefore, the downregulation of lncRNA-SNHG1 after menopause could serve as a valuable biomarker for the diagnosis and treatment of postmenopausal OP [81]. Another study revealed differences in the transcriptome between healthy individuals and patients with OP in a Chinese population. The *DOCK4* gene and two lncRNAs involved in osteoblast modulation and apoptosis, NONHSAT122777.2 and NONHSAT122778.2 were reported. An analysis evaluating the diagnostic value of these lncRNAs showed AUC values of 87.5% for *DOCK4*, 85% for NONHSAT122777.2, and 87.5% for NONHSAT122778.2. Therefore, the authors suggest that these transcripts could be potential therapeutic targets for pharmacological interventions and gene therapies aimed at modulating their expression to mitigate the progression of OP and improve patient outcomes [82].

## 5. Conclusions

Bone remodeling is a mechanism controlled by the bone formation and resorption balance. However, this process involves the activation of numerous biological processes. This review has summarized the current knowledge on lncRNAs; their OP-associated functions; their involvement in the differentiation of osteoclasts, osteoblasts, and osteocytes; and the molecular mechanisms that mediate these effects. Many lncRNAs interact with RNPs or miRNAs to control the expression of their target genes, thereby influencing numerous signaling pathways involved in bone formation and resorption. Furthermore, although lncRNAs hold great potential in predicting, prognosis, and diagnosing OP, their regulatory capacity to target proteins remains largely unknown.

Further research is necessary to explore the transduction and communication pathways of lncRNAs, along with their intracellular signaling, and both direct or indirect targets involved in oxidative stress, adverse effects, and microenvironmental changes during bone remodeling, to identify strategies for maintaining a balance between bone resorption and formation. Additionally, it is crucial to focus on treatments that enable specific lncRNAs to function efficiently at particular locations and at different stages of bone diseases.

A deeper understanding of lncRNAs has the potential to enhance their clinical utility as biomarkers for the early detection of bone-metabolism-related diseases including OP. In this regard, the knowledge of lncRNAs is still at an early stage, and further studies are needed to establish safety, efficacy, and targeted delivery systems that allow their use as modulators. Advancements in our understanding of the molecules involved in the pathophysiology of OP will not only provide tools for the earlier and more accurate detection of the disease but also pave the way for the development of more effective and personalized therapies.

## Figures and Tables

**Table 1:** LncRNAs involved in osteoclastogenesis in cell lines and animal models.

LncRNA ID	Study Model	Target	Effects on Bone Remodeling	Reference
NCRNA00047 (MALAT1/NEAT2)	Exosomes derived of EPC	miR-124/*ITGB1*	Promotes recruitment and differentiation of osteoclast precursors	[37]
NCRNA00084 (NEAT1)	Mice	miR-7b/*PTK2* *BMP1*/miR-29b-3p *DDX5*/Wnt/β-catenin	Promotes osteoclastogenesis	[38,39,40]
AK077216	Mice	*NIP45*	Promotes osteoclastogenesis	[41]
NCRNA00194 (NRON)	Mice	*NFATC1*	Inhibits osteoclastogenesis	[42]
NR4A1AS	Mice	*UPF1*	Control of migration and recruitment of osteoclast precursors	[43]
HGNC:54188 (Bmncr)	RAW264.7	*RANKL*	Inhibits osteoclastogenesis	[44]
HGNC:54188 (Bmncr)	RAW264.7	*RANKL*	Promotes osteoclastogenesis	[44]
FLJ38860 (SNHG15)	HFOB1.19	miR-14/*RANK*/*RANKL*	Regulates osteoclastogenesis	[45]
LncRNA-Jack3	RAW264.7	*Jack3*/*Nfatc1*/*Ctsk*	Regulates osteoclastogenesis	[46]
HGNC:482 (ANCR)	HFOB1.19	*RUNX2*	Inhibits osteoclastogenesis	[47]
DXS399E (XIST)	RAW264.7/BMMs	*FUS*/*SPHK1*/*S1P*/*ERK*	Promotes osteoclastogenesis	[48]
lncRNA-NONMMUT000375.2	RAW264.7/MC3T3-E1	*Bcl2*, *Wnt11*, *TGFB1*, and *Pdk1*	Promotes osteoclastogenesis	[49]
NCRNA00080 (TUG1)	VECs	miR-204-5p/*PTEN*	Promotes osteoclastogenesis	[50]

MALAT1: Metastasis-Associated Lung Adenocarcinoma Transcript 1, NEAT1: Nuclear Paraspeckle Assembly Transcript 1, NEAT2: Nuclear Paraspeckle Assembly Transcript 2, *EPC*: Endothelial Progenitor Cell, *ITGB1*: Integrin Subunit Beta 1, *PTK2*: Protein Tyrosine Kinase 2, *BMP1*: Bone Morphogenetic Protein 1, *DDX5*: DEAD-Box Helicase 5, *NFATC1*: Nuclear Factor Of Activated T Cells 1, *UPF1*: Up-Frameshift Suppressor 1, *RANKL*: Receptor Activator for Nuclear Factor κ B Ligand, *Ctsk:* Cathepsin K, *RUNX2*: RUNX Family Transcription Factor 2, *FUS*: FUS RNA Binding Protein, *SPHK1*: Sphingosine Kinase 1, *S1P*: Esfingosina-1-fosfato, *ERK*: Extracellular-Signal-Regulated Kinase, *Bcl2*: Bcl2 Apoptosis Regulator, *Wnt11*: Wnt Family Member 11, *TGFB1*: Transforming Growth Factor Beta 1, *Pdk1*: Pyruvate Dehydrogenase Kinase 1, *PTEN*: Phosphatase And Tensin Homolog, and VECs: Valve Endothelial Cells.

**Table 2:** LncRNAs involved in osteoblastogenesis in humans, cell lines and animal models.

LncRNA ID	Study Model	Target	Effects on Bone Remodeling	Reference
HGNC:29665 (MALAT1)	ICAV	miR-204/*SMAD4*	Promotes differentiation of osteoblasts and osteocytes	[58]
NCRNA00057 (SNHG1)	Osteoblasts and serum samples	*OCN* *ALP*	Inhibits osteoblast differentiation	[59]
HGNC:30080 (POM121L9P)	MSC	miR-503-5p/*SORBS1*	Inhibits osteoblast differentiation	[60]
HGNC:7737 (NEFH)	Serum samples	miR-155/*PTEN*	Promotes differentiation of osteoblasts	[61]
NCRNA00213 (HOTTIP)	MSC	Wnt/β-catenin signaling pathway	Promotes differentiation of osteoblasts	[62]
HGNC:51599 (NKILA)	MSC	*RXFP1*/*PI3K*-*AKT*/*NF-kB*	Promotes differentiation of osteoblasts	[63]
NCRNA00066 (MIAT)	Serum samples	miR-150-5p	Inhibits osteoblast differentiation	[64]
NCRNA00012 (KCNQ1OT1)	MSC	miR-205-5p/*RICTOR* *OPN*, *RUNX2*, *OCN*	Promotes differentiation of osteoblasts	[65]
NCRNA00008 (H19)	MC3T3-E1	miR-185-5p/*IGF1*	Modulating matrix mineralization of osteoblasts	[66]
HGNC:54521 (POIR)	MSC	miR-182/*FoxO1*	Promotes differentiation of osteoblasts	[67]
HGNC:31337 (HCG18)	NP	miR-146a-5p/*TRAF6*/*NF-kB*	Regulates differentiation of osteoblasts	[68]
HGNC:43748 (HOXA-AS3)	MSC	*EZH2*/*Runx2*/H3K27me3	Inhibits osteoblast differentiation	[69]
lncRUNX2-AS1	MSC	*RUNX2*	Inhibits osteoblast differentiation	[70]
MODR	MSC	miR-454	Promotes differentiation of osteoblasts	[71]
HGNC:43015 (HIF1A-AS2)	hpPDLSC	*HIF-1α*	Osteogenic differentiation of periodontal ligament cells	[72]
TCONS_00041960	rBMC	miR-204-5p/miR-125a-3p/*Runx2*/*GILZ*	Promotes/osteogenesis	[73]

MALAT1: Metastasis-Associated Lung Adenocarcinoma Transcript, ICAV: Interstitial Cells of Aortic Valve, *ALP*: Alkaline phospatase, *OCN*: Osteocalcin, *OPN*: Osteopontin, *RUNX2*: RUNX Family Transcription Factor, MSC: Mesenchymal Stem Cell, *PTEN*: Phosphatase and Tensin Homolog, *SORBS1*: Sorbin and SH3 domain-containing 1, *RICTOR*: RPTOR-Independent Companion of MTOR Complex 2, NP: Nucleus Pulposus, hpPDLSC: Human Periodontal Ligament Stem Cell, and rBMC: Rat Marrow Mesenchymal Stem Cell. SMAD4: SMAD Family Member 4, SNHG1: Small Nucleolar RNA Host Gene 1, *RXFP1*: Relaxin Family Peptide Receptor 1, *NF-kB*: Nuclear Factor Kappa B, *IGF1*: Insulin Like Growth Factor 1, *FoxO1*: Forkhead Box O1, *TRAF6*: TNF Receptor Associated Factor 6, *EZH2*: Enhancer Of Zeste 2 Polycomb Repressive Complex 2 Subunit, *HIF1α*: Hypoxia Inducible Factor 1 Subunit Alpha, NEFH: Neurofilament Heavy Chain, HOTTIP: HOXA Distal Transcript Antisense RNA, NKILA: NF-KappaB Interacting LncRNA, MIAT: Myocardial Infarction Associated Transcript, POIR: LncRNA Periodontal Mesenchymal Stem Cell Osteogenesis Related, HCG18: HLA Complex Group 18, HOXA-AS3: HOXA Cluster Antisense RNA 3, HIF1A-AS2: HIF1A Antisense RNA 2.

## References

[1] Silva A.M., Moura S.R., Teixeira J.H., Barbosa M.A., Santos S.G., Almeida M.I. (2019). Long noncoding RNAs: A missing link in osteoporosis. Bone Res..

[2] Odén A., McCloskey E.V., Kanis J.A., Harvey N.C., Johansson H. (2015). Burden of high fracture probability worldwide: Secular increases 2010–2040. Osteoporos. Int..

[3] Williams S.A., Daigle S.G., Weiss R., Wang Y., Arora T., Curtis J.R. (2021). Economic Burden of Osteoporosis-Related Fractures in the US Medicare Population. Ann. Pharmacother..

[4] Aziziyeh R., Amin M., Habib M., Garcia Perlaza J., Szafranski K., McTavish R.K., Disher T., Lüdke A., Cameron C. (2019). The burden of osteoporosis in four Latin American countries: Brazil, Mexico, Colombia, and Argentina. J. Med. Econ..

[5] Bolamperti S., Villa I., Rubinacci A. (2022). Bone remodeling: An operational process ensuring survival and bone mechanical competence. Bone Res..

[6] Arias C.F., Herrero M.A., Echeverri L.F., Oleaga G.E., López J.M. (2018). Bone remodeling: A tissue-level process emerging from cell-level molecular algorithms. PLoS ONE.

[7] Del Fattore A., Cappariello A., Teti A. (2008). Genetics, pathogenesis and complications of osteopetrosis. Bone.

[8] Marini F., Cianferotti L., Brandi M.L. (2016). Epigenetic Mechanisms in Bone Biology and Osteoporosis: Can They Drive Therapeutic Choices?. Int. J. Mol. Sci..

[9] Huynh N.P., Anderson B.A., Guilak F., McAlinden A. (2017). Emerging roles for long noncoding RNAs in skeletal biology and disease. Connect. Tissue Res..

[10] Choudhuri S. (2023). Long noncoding RNAs: Biogenesis, regulation, function, and their emerging significance in toxicology. Toxicol. Mech. Methods..

[11] Liu Y., Ding W., Yu W., Zhang Y., Ao X., Wang J. (2021). Long non-coding RNAs: Biogenesis, functions, and clinical significance in gastric cancer. Mol. Ther. Oncolytics.

[12] Aurilia C., Donati S., Palmini G., Miglietta F., Iantomasi T., Brandi M.L. (2021). The Involvement of Long Non-Coding RNAs in Bone. Int. J. Mol. Sci..

[13] Chen R., Wang G., Zheng Y., Hua Y., Cai Z. (2017). Long non-coding RNAs in osteosarcoma. Oncotarget.

[14] Chodurska B., Kunej T. (2025). Long non-coding RNAs in humans: Classification, genomic organization and function. Noncoding RNA Res..

[15] Chen L.L., Kim V.N. (2024). Small and long non-coding RNAs: Past, present, and future. Cell.

[16] Nojima T., Proudfoot N.J. (2022). Mechanisms of lncRNA biogenesis as revealed by nascent transcriptomics. Nat. Rev. Mol. Cell Biol..

[17] He Y., Chen Y. (2021). The potential role of lncRNAs in osteoporosis. J. Bone Min. Metab..

[18] Mercer T.R., Dinger M.E., Mattick J.S. (2009). Long non-coding RNAs: Insights into functions. Nat. Rev. Genet..

[19] Sebastian-delaCruz M., Gonzalez-Moro I., Olazagoitia-Garmendia A., Castellanos-Rubio A., Santin I. (2021). The Role of lncRNAs in Gene Expression Regulation through mRNA Stabilization. Noncoding RNA.

[20] Nekrutenko A., Li W.H. (2001). Transposable elements are found in a large number of human protein-coding genes. Trends Genet..

[21] Dhanoa J.K., Sethi R.S., Verma R., Arora J.S., Mukhopadhyay C.S. (2018). Long non-coding RNA: Its evolutionary relics and biological implications in mammals: A review. J. Anim. Sci. Technol..

[22] Ahmad P., Bensaoud C., Mekki I., Rehman M.U., Kotsyfakis M. (2021). Long Non-Coding RNAs and Their Potential Roles in the Vector-Host-Pathogen Triad. Life.

[23] Graf J., Kretz M. (2020). From structure to function: Route to understanding lncRNA mechanism. Bioessays.

[24] Zhang X., Wang W., Zhu W., Dong J., Cheng Y., Yin Z., Shen F. (2019). Mechanisms and Functions of Long Non-Coding RNAs at Multiple Regulatory Levels. Int. J. Mol. Sci..

[25] Wang W., Min L., Qiu X., Wu X., Liu C., Ma J., Zhang D., Zhu L. (2021). Biological Function of Long Non-coding RNA (LncRNA) Xist. Front. Cell Dev. Biol..

[26] Thomson D.W., Dinger M.E. (2016). Endogenous microRNA sponges: Evidence and controversy. Nat. Rev. Genet..

[27] Davidovich C., Cech T.R. (2015). The recruitment of chromatin modifiers by long noncoding RNAs: Lessons from PRC2. RNA.

[28] Luo J., Qu L., Gao F., Lin J., Liu J., Lin A. (2021). LncRNAs: Architectural Scaffolds or More Potential Roles in Phase Separation. Front. Genet..

[29] Hou Y., Zhang R., Sun X. (2019). Enhancer LncRNAs Influence Chromatin Interactions in Different Ways. Front. Genet..

[30] Dou C., Cao Z., Yang B., Ding N., Hou T., Luo F., Kang F., Li J., Yang X., Jiang H. (2016). Changing expression profiles of lncRNAs, mRNAs, circRNAs and miRNAs during osteoclastogenesis. Sci. Rep..

[31] Cong C., Tian J., Gao T., Zhou C., Wang Y., Cui X., Zhu L. (2020). lncRNA GAS5 Is Upregulated in Osteoporosis and Downregulates miR-21 to Promote Apoptosis of Osteoclasts. Clin. Interv. Aging.

[32] Yu H., Zhou W., Yan W., Xu Z., Xie Y., Zhang P. (2019). LncRNA CASC11 is upregulated in postmenopausal osteoporosis and is correlated with TNF-α. Clin. Interv. Aging.

[33] Du Y.J., Yu Q.Q., Zheng X.F., Wang S.P. (2020). LncRNA TUG1 positively regulates osteoclast differentiation by targeting v-maf musculoaponeurotic fibrosarcoma oncogene homolog B. Autoimmunity.

[34] Li C., Wang S., Xing Z., Lin A., Liang K., Song J., Hu Q., Yao J., Chen Z., Park P.K. (2017). A ROR1-HER3-lncRNA signalling axis modulates the Hippo-YAP pathway to regulate bone metastasis. Nat. Cell Biol..

[35] Ling L., Hu H.L., Liu K.Y., Ram Y.I., Gao J.L., Cao Y.M. (2019). Long noncoding RNA MIRG induces osteoclastogenesis and bone resorption in osteoporosis through negative regulation of miR-1897. Eur. Rev. Med. Pharmacol. Sci..

[36] Liu W., Li Z., Cai Z., Xie Z., Li J., Li M., Cen S., Tang S., Zheng G., Ye G. (2020). Perfiles de expresión de LncRNA-mRNA y redes funcionales en diferenciación osteoclasta. J. Cell. Mol. Med..

[37] Cui Y., Fu S., Sun D., Xing J., Hou T., Wu X. (2019). EPC-derived exosomes promote osteoclastogenesis through LncRNA-MALAT1. J. Cell. Mol. Med..

[38] Dou C., Zhang C., Kang F., Yang X., Jiang H., Bai Y., Xiang J., Xu J., Dong S. (2014). MiR-7b directly targets DC-STAMP causing suppression of NFATc1 and c-Fos signaling during osteoclast fusion and differentiation. Biochim. Biophys. Acta.

[39] Zhang Y., Chen B., Li D., Zhou X., Chen Z. (2019). LncRNA NEAT1/miR-29b-3p/BMP1 axis promotes osteogenic differentiation in human bone marrow-derived mesenchymal stem cells. Pathol. Res. Pract..

[40] Zhang M., Weng W., Zhang Q., Wu Y., Ni S., Tan C., Xu M., Sun H., Liu C., Wei P. (2018). The lncRNA NEAT1 activates Wnt/β-catenin signaling and promotes colorectal cancer progression via interacting with DDX5. J. Hematol. Oncol..

[41] Liu C., Cao Z., Bai Y., Dou C., Gong X., Liang M., Dong R., Quan H., Li J., Dai J. (2019). LncRNA AK077216 promotes RANKL-induced osteoclastogenesis and bone resorption via NFATc1 by inhibition of NIP45. J. Cell Physiol..

[42] Zhang R., Li J., Li G., Jin F., Wang Z., Yue R., Wang Y., Wang X., Sun Y. (2020). LncRNA Nron regulates osteoclastogenesis during orthodontic bone resorption. Int. J. Oral. Sci..

[43] Scholtysek C., Ipseiz N., Böhm C., Krishnacoumar B., Stenzel M., Czerwinski T., Palumbo-Zerr K., Rothe T., Weidner D., Klej A. (2018). NR4A1 Regulates Motility of Osteoclast Precursors and Serves as Target for the Modulation of Systemic Bone Turnover. J. Bone Min. Miner. Res..

[44] Chen R.S., Zhang X.B., Zhu X.T., Wang C.S. (2019). LncRNA Bmncr alleviates the progression of osteoporosis by inhibiting RANML-induced osteoclast differentiation. Eur. Rev. Med. Pharmacol. Sci..

[45] Liu K., Hou Y., Liu Y., Zheng J. (2017). LncRNA SNHG15 contributes to proliferation, invasion and autophagy in osteosarcoma cells by sponging miR-141. J. Biomed. Sci..

[46] Lee C.P., Huang Y.N., Nithiyanantham S., Huang C.M., Ko Y.C. (2019). LncRNA-Jak3: Jak3 coexpressed pattern regulates monosodium urate crystal-induced osteoclast differentiation through Nfatc1/Ctsk expression. Environ. Toxicol..

[47] Zhu L., Xu P.C. (2013). Downregulated LncRNA-ANCR promotes osteoblast differentiation by targeting EZH2 and regulating Runx2 expression. Biochem. Biophys. Res. Commun..

[48] Zhang D.W., Wang H.G., Zhang K.B., Guo Y.Q., Yang L.J., Lv H. (2022). LncRNA XIST facilita la diferenciación osteoclata mediada por S1P mediante la interacción con FUS. J. Bone Min. Metab..

[49] Xu J., Li D., Cai Z., Sun H., Su B., Qiu M., Ma R. (2020). Exosomal lncRNAs NONMMUT000375.2 and NONMMUT071578.2 derived from titanium particle treated RAW264.7 cells regulate osteogenic differentiation of MC3T3-E1 cells. J. Biomed. Mater. Res. A..

[50] Yu C., Li L., Xie F., Guo S., Liu F., Dong N., Wang Y. (2018). LncRNA TUG1 sponges miR-204-5p to promote osteoblast differentiation through upregulating Runx2 in aortic valve calcification. Cardiovasc. Res..

[51] Fatica A., Bozzoni I. (2014). Long non-coding RNAs: New players in cell differentiation and development. Nat. Rev. Genet..

[52] Jiang S.Y., Miao Y.X., Hirokazu T., Zhu S.Z., Lu J.S. (2019). Effects of lncRNA DANCR on proliferation and differentiation of osteoblasts by regulating the Wnt/β-catenin pathway. Eur. Rev. Med. Pharmacol. Sci..

[53] Wang C.G., Hu Y.H., Su S.L., Zhong D. (2020). LncRNA DANCR and miR-320a suppressed osteogenic differentiation in osteoporosis by directly inhibiting the Wnt/β-catenin signaling pathway. Exp. Mol. Med..

[54] Wei B., Wei W., Zhao B., Guo X., Liu S. (2017). Long non-coding RNA HOTAIR inhibits miR-17-5p to regulate osteogenic differentiation and proliferation in non-traumatic osteonecrosis of femoral head. PLoS ONE.

[55] Li X.G., Liu S.C., Qiao X.F., Kong Y., Liu J.G., Peng X.M., Wang Y.X., Abdulkarim Mohammed Al-Mohana R.A. (2019). LncRNA MEG3 promotes proliferation and differentiation of osteoblasts through Wnt/β-catenin signaling pathway. Eur. Rev. Med. Pharmacol. Sci..

[56] Che M., Gong W., Zhao Y., Liu M. (2020). Long noncoding RNA HCG18 inhibits the differentiation of human bone marrow-derived mesenchymal stem cells in osteoporosis by targeting miR-30a-5p/NOTCH1 axis. Mol. Med..

[57] Han Y., Yang Q., Huang Y., Jia L., Zheng Y., Li W. (2022). Long non-coding RNA SNHG5 promotes the osteogenic differentiation of bone marrow mesenchymal stem cells via the miR-212-3p/GDF5/SMAD pathway. Stem Cell Res. Ther..

[58] Xiao X., Zhou T., Guo S., Guo C., Zhang Q., Dong N., Wang Y. (2017). LncRNA MALAT1 sponges miR-204 to promote osteoblast differentiation of human aortic valve interstitial cells through up-regulating Smad4. Int. J. Cardiol..

[59] Pan K., Lu Y., Cao D., Peng J., Zhang Y., Li X. (2024). Long Non-coding RNA SNHG1 Suppresses the Osteogenic Differentiation of Bone Marrow Mesenchymal Stem Cells by Binding with HMGB1. Biochem. Genet..

[60] Xu Y., Xin R., Sun H., Long D., Li Z., Liao H., Xue T., Zhang Z., Kang Y., Mao G. (2021). Long Non-coding RNAs LOC100126784 and POM121L9P Derived from Bone Marrow Mesenchymal Stem Cells Enhance Osteogenic Differentiation via the miR-503-5p/SORBS1 Axis. Front. Cell Dev. Biol..

[61] Ming Y., Liu Z.P. (2021). Overexpression of lncRNA-NEF regulates the miR-155/PTEN axis to inhibit adipogenesis and promote osteogenesis. Kaohsiung J. Med. Sci..

[62] Liu R., Li Z., Song E., Hu P., Yang Q., Hu Y., Liu H., Jin A. (2020). LncRNA HOTTIP enhances human osteogenic BMSCs differentiation via interaction with WDR5 and activation of Wnt/β-catenin signalling pathway. Biochem. Biophys. Res. Commun..

[63] Zhang Y., Cao X., Li P., Fan Y., Zhang L., Ma X., Sun R., Liu Y., Li W. (2020). LncRNA NKILA integrates RXFP1/AKT and NF-κB signalling to regulate osteogenesis of mesenchymal stem cells. J. Cell. Mol. Med..

[64] Wang F., Deng H., Chen J., Wang Z., Yin R. (2022). LncRNA MIAT can regulate the proliferation, apoptosis, and osteogenic differentiation of bone marrow-derived mesenchymal stem cells by targeting miR-150-5p. Bioengineered.

[65] Yang J.J., Peng W.X., Zhang M.B. (2022). LncRNA KCNQ1OT1 promotes osteogenic differentiation via miR-205-5p/RICTOR axis. Exp. Cell Res..

[66] Wu Y., Jiang Y., Liu Q., Liu C.Z. (2019). lncRNA H19 promotes matrix mineralization through up-regulating IGF1 by sponging miR-185-5p in osteoblasts. BMC Mol. Cell Biol..

[67] Wang L., Wu F., Song Y., Li X., Wu Q., Duan Y., Jin Z. (2016). Long noncoding RNA related to periodontitis interacts with miR-182 to upregulate osteogenic differentiation in periodontal mesenchymal stem cells of periodontitis patients. Cell Death Dis..

[68] Xi Y., Jiang T., Wang W., Yu J., Wang Y., Wu X., He Y. (2017). Long non-coding HCG18 promotes intervertebral disc degeneration by sponging miR-146a-5p and regulating TRAF6 expression. Sci. Rep..

[69] Zhu X.X., Yan Y.W., Chen D., Ai C.Z., Lu X., Xu S.S., Jiang S., Zhong G.S., Chen D.B., Jiang Y.Z. (2016). Long non-coding RNA HoxA-AS3 interacts with EZH2 to regulate lineage commitment of mesenchymal stem cells. Oncotarget.

[70] Li B., Xu H., Han H., Song S., Zhang X., Ouyang L., Qian C., Hong Y., Qiu Y., Zhou W. (2018). Exosome-mediated transfer of lncRUNX2-AS1 from multiple myeloma cells to MSCs contributes to osteogenesis. Oncogene.

[71] Weng J., Peng W., Zhu S., Chen S. (2017). Long Noncoding RNA Sponges miR-454 to Promote Osteogenic Differentiation in Maxillary Sinus Membrane Stem Cells. Implant. Dent..

[72] Chen D., Wu L., Liu L., Gong Q., Zheng J., Peng C., Deng J. (2017). Comparison of HIF1A AS1 and HIF1A AS2 in regulating HIF 1α and the osteogenic differentiation of PDLCs under hypoxia. Int. J. Mol. Med..

[73] Shang G., Wang Y., Xu Y., Zhang S., Sun X., Guan H., Zhao X., Wang Y., Li Y., Zhao G. (2018). Long non-coding RNA TCONS_00041960 enhances osteogenesis and inhibits adipogenesis of rat bone marrow mesenchymal stem cell by targeting miR-204-5p and miR-125a-3p. J. Cell Physiol..

[74] Fu L., Peng S., Wu W., Ouyang Y., Tan D., Fu X. (2019). LncRNA HOTAIRM1 promotes osteogenesis by controlling JNK/AP-1 signalling-mediated RUNX2 expression. J. Cell. Mol. Med..

[75] Yu X., Rong P.Z., Song M.S., Shi Z.W., Feng G., Chen X.J., Shi L., Wang C.H., Pang Q.J. (2021). lncRNA SNHG1 induced by SP1 regulates bone remodeling and angiogenesis via sponging miR-181c-5p and modulating SFRP1/Wnt signaling pathway. Mol. Med..

[76] Arai M., Ochi H., Sunamura S., Ito N., Nangaku M., Takeda S., Sato S. (2023). A Novel Long Noncoding RNA in Osteocytes Regulates Bone Formation through the Wnt/β-Catenin Signaling Pathway. Int. J. Mol. Sci..

[77] Zhao Y., Ning J., Teng H., Deng Y., Sheldon M., Shi L., Martinez C., Zhang J., Tian A., Sun Y. (2024). Long noncoding RNA Malat1 protects against osteoporosis and bone metastasis. Nat. Commun..

[78] Ouyang X., Ding Y., Yu L., Xin F., Yang X. (2022). LncRNA TUG regulates osteogenic differentiation of bone marrow mesenchymal stem cells via miRNA-204/SIRT 1. J. Musculoskelet. Neuronal Interact..

[79] Qian T.Y., Wan H., Huang C.Y., Hu X.J., Yao W.F. (2022). Plasma LncRNA MALAT1 Expressions Are Negatively Associated with Disease Severity of Postmenopausal Osteoporosis. Lab. Med..

[80] Ma X., Guo Z., Gao W., Wang J., Liu Y., Gao F., Sun S., Zhou X., Yang Z., Zheng W. (2019). LncRNA-NEF is downregulated in postmenopausal osteoporosis and is related to course of treatment and recurrence. J. Int. Med. Res..

[81] Huang S., Zhu X., Xiao D., Zhuang J., Liang G., Liang C., Zheng X., Ke Y., Chang Y. (2019). LncRNA SNHG1 was down-regulated after menopause and participates in postmenopausal osteoporosis. Biosci. Rep..

[82] Wu C., Wang C., Xiao B., Li S., Sheng Y., Wang Q., Tao J., Zhang Y., Jiang X. (2024). Integration analysis of lncRNA and mRNA expression data identifies DOCK4 as a potential biomarker for elderly osteoporosis. BMC Med. Genom..

